# Molecular imaging in heart failure patients

**DOI:** 10.1007/s40336-013-0034-y

**Published:** 2013-10-09

**Authors:** Nagara Tamaki, Yuji Kuge, Keiichiro Yoshinaga

**Affiliations:** 1 Department of Nuclear Medicine, Graduate School of Medicine, Hokkaido University, N-15, W-7, Kita-ku, Sapporo, 060-8538 Japan; 2 Central Institute of Isotope Science, Hokkaido University, Sapporo, Japan

**Keywords:** Radionuclide imaging, Positron emission tomography, Molecular imaging, Heart failure, Adrenergic neuronal imaging, Receptor imaging

## Abstract

This review focuses on molecular imaging using various radioligands for the tissue characterization of patients with heart failure. ^123^I-labeled metaiodobenzylguanidine (MIBG), as a marker of adrenergic neuron function, plays an important role in risk stratification in heart failure and may be useful for predicting fatal arrhythmias that may require implantable cardioverter-defibrillator treatment. MIBG has also been used for monitoring treatment effects under various medications. Various positron emission tomography (PET) radioligands have been introduced for the quantitative assessment of presynaptic and postsynaptic neuronal function in vivo. ^11^C-hydroxyephedrine, like MIBG, has potential for assessing the severity of heart failure. Our PET study using the β-receptor antagonist ^11^C-CGP 12177 in patients with heart failure showed a reduction of β-receptor density, indicating downregulation, in most of the patients. More studies are needed to confirm the clinical utility of these molecular imaging modalities for the management of heart failure patients.

## Introduction

Heart failure is a major cause of mortality and morbidity. The treatment of heart failure patients has been found to entail tremendous medical costs in many countries [1, 2]. Although heart failure treatment has improved greatly, the 5-year mortality rate continues to be 40–50 % [2, 3]. Myocardial infarction (MI) is recognized as a prime cause of systolic heart failure [4, 5]. An acute MI will result in changes in left ventricular (LV) size, shape, and wall thickness that involve the infarcted and non-infarcted regions; these changes are referred to as “LV remodeling” [6–9]. Various myocardial disorders, including dilated cardiomyopathy, also cause severe LV dilatation with heart failure.

A number of non-invasive cardiovascular imaging modalities are currently used to assess the severity of heart failure. Among them, molecular imaging has recently been a focus of interest on account of its ability to characterize tissues involved in myocardial disorders at different levels: molecular, subcellular, and cellular [10–12]. Molecular imaging promises to not only deepen our understanding of already known biological processes, but also to uncover unknown molecular and cellular events that are at the center of the initiation and evolution of disease. Compared to traditional in vitro tissue/cell culture and ex vivo animal studies, molecular imaging permits the non-invasive and repetitive imaging of targeted biological processes at both cellular and subcellular levels in live organs. Molecular imaging thus offers a means to visualize specific abnormal biological processes for diagnosis and treatment.

Radionuclide imaging, using either positron emission tomography (PET) or single-photon emission computed tomography (SPECT), has been the modality most commonly used for human molecular imaging, mainly due to its quite high sensitivity to radiolabeled ligands with nano- or micro-order levels compared to other methods of molecular imaging. Even though molecular imaging has low spatial and temporal resolutions and high background activity and radiation, radionuclide imaging has been safely used in a wide range of clinical applications, including evaluations of various disease processes.

## Imaging autonomic functions

As a result of advances in vivo biochemical imaging using radionuclide techniques, the modulation of functional and electrophysiological properties of the heart by the autonomic nervous system has become a focus of interest in the field of cardiovascular research. Cardiac neuronal function is compromised in various cardiac diseases, including congestive heart failure, ischemia, arrhythmia, and some types of cardiomyopathy [13]. Tracer approaches are considered uniquely suited for radionuclide imaging-based in vivo characterization of neuronal function in the myocardium.

The autonomic nervous system consists of two main parts: sympathetic and parasympathetic innervations. Their major transmitters are norepinephrine and acetylcholine, respectively, which define the stimulatory and inhibitory physiological effects of each system. Sympathetic innervation originates mainly from the right and left stellate ganglia, which provide the sympathetic nerves to form the cardiac plexus of the heart. The sympathetic nerve fibers travel parallel to the vascular structures on the epicardial surface of the heart and then penetrate the underlying myocardium in a manner similar to that seen in the coronary vessels [14]. As regard to tissue levels of norepinephrine, the mammalian heart is characterized by dense adrenergic innervation with a norepinephrine concentration gradient from the atria to the base of the heart and from the base to the apex of the left ventricle [15, 16].

Parasympathetic innervation, by contrast, originates from the medulla and passes through the right and left vagal nerves, which further divide into the superior and inferior cardiac nerves. Parasympathetic nerve fibers primarily modulate sinoatrial nodal and atrioventricular nodal function and innervate the atria, whereas, vagal fibers to the ventricles are rather sparse [17]. These autonomic nervous systems are involved in the synthesis and storage of neurotransmitters and their release, reuptake, metabolism, and interaction with presynaptic and postsynaptic receptor sites.

**Fig. 1:**
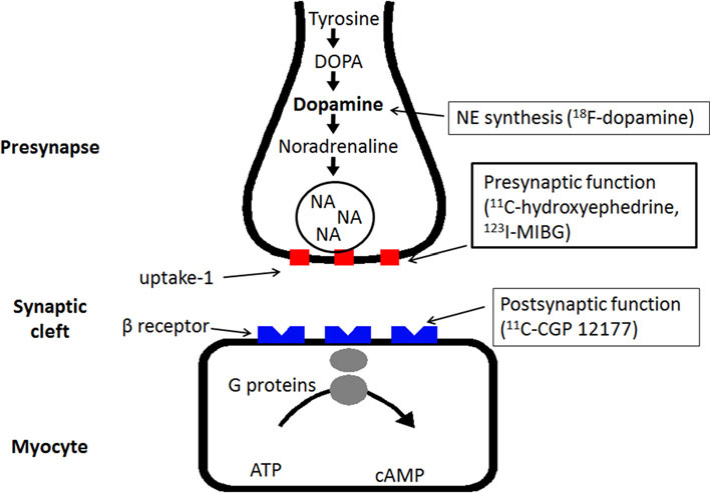
Schema of myocardial nerve terminals and various radioligands used to probe adrenergic functions (Color figure online)

There are a number of radiotracers that can be used to probe each step of autonomic neuronal functions. Table 1 shows representative radiotracers used to probe sympathetic and parasympathetic nerve functions, both presynaptic and postsynaptic. Figure 1 illustrates a number of adrenergic presynaptic and postsynaptic (receptor) functions, indicating the radiopharmaceuticals that have been used to probe those functions. For example, Eisenhoffer et al. [18–20] used radiolabeled norepinephrine for the assessment of sympathetic nerve function and norepinephrine kinetics.

**Table 1:** Radiotracers used for the evaluation of autonomic nervous system functions

	Sympathetic nerves	Parasympathetic nerves
Presynaptic	^123^I-metaiodobenzylguanidine (MIBG)	^123^I-iodobenzoversamicol
	^11^C-hydroxyephedrine	^18^F-fluorobenzyl-benzovesamicol
	^18^F-metaraminol	
	^18^F-dopamine	
	^11^C-threohydroxyepinephrine	
	^11^C-epinephrine	
Postsynaptic	I-iodocyanopindolol (ICYP)	^123^I-quinuclidinyl benzylate (QNB)
	^11^C-practolol	^11^C-methyl QNB
	^11^C-propranolol	
	^11^C-CGP 12177	
	^11^C-prazocin

Among these radiopharmaceuticals, the norepinephrine analog metaiodobenzylguanidine (MIBG) has been widely used in experimental and clinical studies for sympathetic nerve imaging. In the early 1980s, University of Michigan researchers developed ^131^I MIBG as a norepinephrine analog for the selective mapping of sympathetic nerve endings in the heart [21, 22]. ^123^I-labeled MIBG is now commonly used for cardiac imaging.

### Fundamental aspects of ^123^I-labeled MIBG

MIBG is an analog of the antihypertensive drug guanethidine and of norepinephrine itself. It is taken up by sympathetic nerves in a similar manner to norepinephrine, but is not metabolized. Most MIBG, following its administration, is actively taken up into neuronal vesicles by means of a Na-dependent specific process (uptake 1), and the remaining MIBG enters the nerve terminals by passive diffusion (uptake 2) [23, 24]. Although uptake 1 accounts for most MIBG distribution in the myocardium, uptake 2 may play a minor role [25, 26]. MIBG is stored by the neurons and is released along with endogenous norepinephrine upon nerve stimulation, but it has a low affinity for postsynaptic adrenergic receptors. Generally, MIBG distribution correlates with that of tissue norepinephrine, but the ability of sympathetic nerve terminals to take up catecholamine is a more sensitive index of nerve function and viability than catecholamine content [27]. Based on the mechanisms of uptake of this SPECT agent, it is recommended to temporarily discontinue medications and substrates that may interfere with norepinephrine uptake, including opioids, antidepressants, reserpine, and others [28].

^123^I-labeled MIBG is used in clinical settings, as it provides better myocardial images than the ^131^I-labeled compound. A clinical study showed that increased sympathetic tone may be associated with increased washout of MIBG [23, 29]. Following the administration of 111–222 MBq (3–6 mCi) of ^123^I-labeled MIBG at rest, myocardial images are usually obtained twice (at 10 min and 4 h) to calculate the myocardial uptake and washout. Sometimes only the delayed images are obtained to estimate delayed myocardial uptake as an index of adrenergic neuron function. In each acquisition, the planar images are obtained in the anterior position to assess the global uptake of MIBG in the myocardium. SPECT imaging can also be added to assess the regional MIBG distribution. The general acquisition time is 3–5 min for planar imaging and 15–30 min for SPECT imaging. A suitable collimator (either a medium-energy collimator or a ^123^I collimator) should be used for ^123^I imaging. Some low-energy collimators are also suitable for ^123^I energy, showing higher sensitivity than medium-energy collimators. Resting administration is most commonly used for the resting state [28, 30].

The radiation dose with the use of 111 MBq of MIBG is 1.2 mGy to the myocardium and 7.9 mGy to the liver. To minimize radiation to the thyroid gland, thyroid blocking is recommended.

**Fig. 2:**
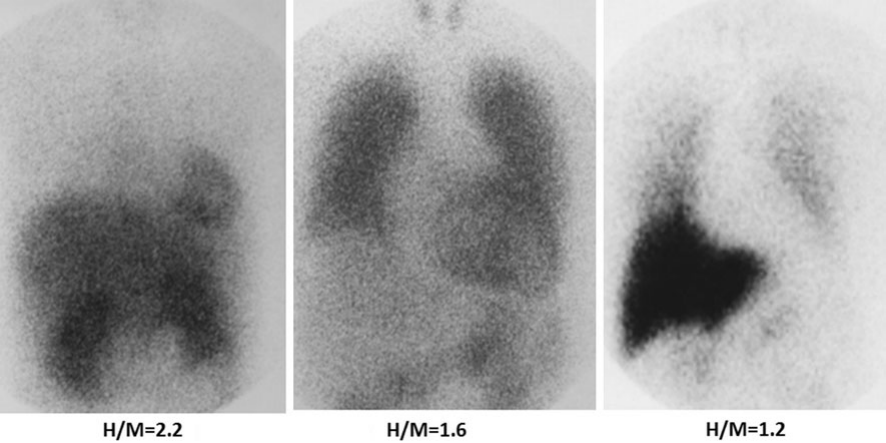
Anterior images at 4 h after ^123^I-labeled MIBG administration in a normal control (*left*), a patient with moderate (*middle*), and a patient with severe heart failure (*right*). The heart-to-mediastinal count ratio (H/M) is a marker of MIBG uptake in the heart

Although ^123^I-labeled MIBG yields high-quality images of myocardial neuronal function, high activity in the liver may be superimposed on myocardial activity in planar images, and thus affect the interpretation of myocardial distribution, particularly in the inferior region. To minimize such superimposition, SPECT is preferred for the assessment of regional MIBG distribution. Planar imaging is used to assess the global uptake of the tracer in the myocardium. Two regions of interest are considered: an irregular region in the whole myocardium and a rectangular region in the upper mediastinum to measure the myocardium-to-mediastinum count rate, which is commonly used as an index of myocardial uptake of MIBG (Fig. 2) [28, 30]. A semi-automatic algorithm for calculating the heart-to-mediastinum (H/M) ratio on MIBG imaging was recently introduced to obtain highly reproducible values [31]. However, there is no established SPECT-based method for estimating or scoring MIBG uptake.

In reports concerning imaging standards and a relevant Japanese database, the normal myocardium-to-mediastinum count rate ranged from 2.0 to 2.7 for the early scan and from 2.1 to 2.9 for the late scan [32, 33], but these values seemed to be dependent on the collimator used for acquisition and the specific activity of the MIBG. Nakajima and colleagues, using different energy collimators, recently developed a calibration phantom as a tool for standardizing MIBG H/M ratio calculation for a normal-values database [34]. This method may be practical for multicenter studies. The washout from the early to the late scan ranged from 21 to 33 %.

In a 1993 clinical study, the normal distribution of MIBG in the myocardium was not quite homogeneous, showing a slight reduction in the inferior region [35]. This heterogeneity seems to be more enhanced with age [36]. In view of this physiological heterogeneity of the tracer distribution in the myocardium, MIBG images should be interpreted cautiously.

### Clinical aspects of ^123^I-labeled MIBG

It is well-known that adrenergic dysfunction plays a key role in heart failure, and the plasma catecholamine level has been recognized as a valuable prognostic tool. Basic studies have indicated that an increased neuronal release of norepinephrine and decreased efficiency of norepinephrine reuptake both contribute to increased cardiac adrenergic drive in congestive heart failure [20]. In addition, decreased vesicular leakage of norepinephrine limits the increase in its cardiac turnover. Thus, analyses of norepinephrine kinetics have a key role in the assessment of the severity of congestive heart failure.

There are a number of reports showing a reduction of MIBG retention in patients with idiopathic dilated cardiomyopathy [37–40]. Schoffer et al. [37] first indicated that the H/M ratio for MIBG is correlated with the plasma norepinephrine level and the left ventricular ejection fraction (LVEF) in patients with dilated cardiomyopathy. Their MIBG results showed that uptake and vesicular storage of norepinephrine were reduced in these patients, similar to the findings in experimental heart failure.

MIBG imaging has long been applied in patients with heart failure in general. A number of papers have highlighted the H/M ratio and/or the washout rate of MIBG as new parameters of heart failure severity and independent prognostic indicators [41–50]. Merlet et al. [41], in 1999, were the first to indicate the prognostic value of the H/M ratio in patients with heart failure. Thereafter, many prognostic studies using this imaging technique were performed in European countries and in Japan; there thus emerged a close association between the H/M ratio and/or the washout rate of MIBG as markers of impaired cardiac adrenergic innervation and mortality in patients with heart failure [41–50]. A meta-analysis of 18 studies including 1,755 patients with heart failure confirmed that heart failure patients showing a reduced H/M ratio on late MIBG imaging had a worse prognosis as compared to those with a normal H/M ratio [51]. A similar meta-analysis was reported from Japan, indicating that both decreased MIBG uptake and its increased washout are indicative of a poor prognosis in heart failure patients [52].

Sudden cardiac death due to fatal arrhythmia is a major healthcare problem. Implantable cardioverter-defibrillator (ICD) treatment has become well established for preventive use in patients at high risk of arrhythmic death. Autonomic dysfunction assessed by MIBG is thought to play an important role in the detection of high-risk ventricular arrhythmia [14, 53, 54]. In particular, MIBG can identify areas of denervation hypersensitivity which may be likely to cause ventricular arrhythmias. A number of pilot studies have indicated MIBG as a potential predictor of ventricular arrhythmias in patients with an ICD [55–60].

Nishisato et al. [57], in a prospective study using both MIBG and resting myocardial perfusion imaging in patients who received ICD treatment, showed that ICD discharge was documented in 30 of the total 60 patients (50 %). They reported that among the various clinical and scintigraphic variables, low H/M ratio on MIBG and a large perfusion defect were the most powerful predictors of ICD discharge in a Cox multivariate analysis. This report was the first to suggest an incremental benefit for the assessment of sympathetic nerve function in combination with myocardial perfusion, and a role for this combined assessment in the risk stratification of patients who may need prophylactic ICD therapy. Both denervation and myocardial scar were important predictors of ICD discharge and fatal arrhythmias. However, it remains unknown whether simple perfusion imaging is enough, or whether both MIBG and perfusion imaging are required to predict such fatal arrhythmias. These authors provided consistent findings showing that ^123^I-labeled MIBG may predict ICD discharge and sudden cardiac death independently of conventional parameters.

Tamaki et al. [61] examined the ability of MIBG imaging to predict sudden cardiac death in comparison with ECG parameters such as T-wave alteration. They found that the MIBG washout rate was independent of LVEF in risk analyses of patients with heart failure. A multivariate Cox analysis suggested that the MIBG washout rate and the LVEF but not ECG parameters were significant and independent predictors of sudden cardiac death. Jacobson et al. [62] reported the results of a prospective MIBG imaging trial of 961 patients with heart failure recruited at 96 sites in North America and Europe. Their ADMIRE-HF study enrolled patients with New York Heart Association (NYHA) Class II and III heart failure and LVEF ≤35 % and revealed that an H/M ratio of <1.6 measured at 4 h after an MIBG administration provided prognostic data beyond that available from the LVEF, B-type natriuretic peptide assay, and NYHA class at the time of enrollment. Their multicenter prospective study confirmed the adrenergic neuronal functional parameter obtained by MIBG scan as an important parameter for predicting sudden death, independent of the commonly used LV functional parameters.

It is important to determine whether adrenergic function can predict the recovery of LV function or an improvement in outcome after treatment in patients with severe heart failure. There are a number of studies indicating that cardiac MIBG uptake improves after β-blocker or other therapy [63–71]. Suwa et al. [63] and Fukuoka et al. [64] both showed improvements of MIBG uptake and washout after β-blocker therapy in patients with dilated cardiomyopathy. The H/M ratio on the delayed images was a good predictor of the response to the β-blocker therapy [63]. Takeishi et al. [65] also reported improvement of MIBG uptake in relation to LVEF recovery after treatment with angiotensin-converting enzyme inhibitor in patients with congestive heart failure. Gerson et al. [66] showed that the H/M ratio improved significantly after carvedilol therapy, especially in patients with an H/M ratio <1.40. Toyama et al. [67] reported favorable changes in symptoms, NYHA functional class, LV function, and the H/M ratio on MIBG imaging following treatment with the β1-blocker metoprolol. Kasama et al. [68] observed a therapeutic effect of carvedilol on MIBG parameters and LV remodeling in patients with dilated cardiomyopathy. These data indicate that MIBG imaging may provide valuable information for the selection and optimization of treatment for congestive heart failure.

### Sympathetic neuronal imaging using PET

**Fig. 3:**
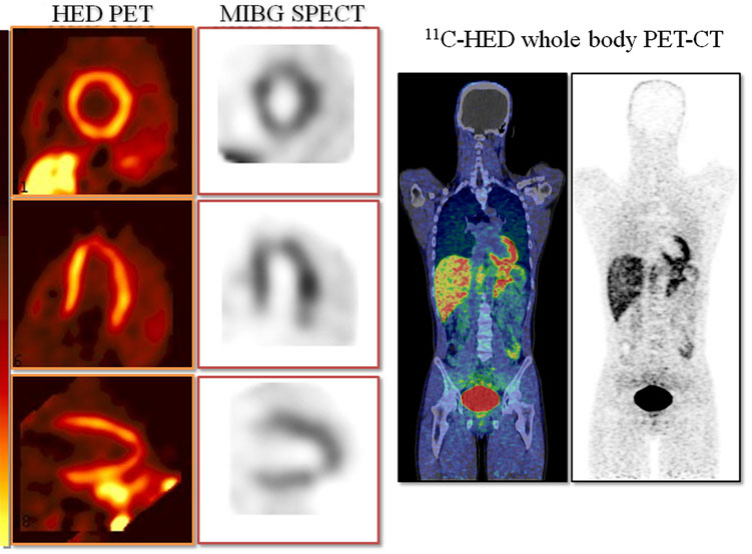
^11^C-hydroxyephedrine (HED) PET and MIBG SPECT of a normal subject. The HED PET obtained better-quality images in the myocardium as compared to the MIBG SPECT (Color figure online)

PET offers great advantages in terms of higher spatial resolution and higher sensitivity with better quantification of tracer concentration compared to the commonly performed SPECT (Fig. 3). With the use of suitable tracer kinetic models, various molecular as well as functional parameters have been estimated in vivo. Various sympathetic neuronal PET tracers have been introduced which closely resemble the endogenous neurotransmitters, and these may allow more detailed analyses of neuronal signaling than MIBG can [72, 73].

^11^C-epinephrine is a radiolabeled true neurotransmitter, and the uptake and storage of this tracer resemble those of norepinephrine [74, 75]. ^11^C-phenylephrine is another PET tracer that is trapped in neuronal vesicles and metabolized by neuronal monoamine oxidase (MAO). Its kinetics may thus reflect MAO metabolism in addition to vesicular leakages [76].

**Fig. 4:**
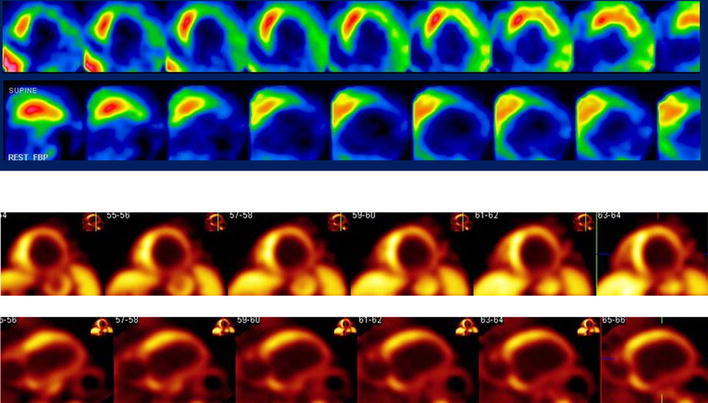
A series of short-axis slices of MIBG SPECT and HED PET images of a patient with heart failure. A moderate reduction of tracer uptake is noted, particularly in the inferior and posterolateral regions (Color figure online)

^11^C-hydroxyephedrine (HED) is the most widely used PET tracer for cardiac neuronal imaging. It has high affinity for presynaptic neuronal catecholamine transporter (uptake 1) without being metabolized by MAO or catechol-*O*-methyl-transferase [77, 78]. Denervation and reinnervation have been investigated using HED in various disorders, such as diabetes [79–81] and heart transplantation [82–84]. A study of patients with heart failure suggested that they had reduced HED uptake, particularly in the apical and inferoapical regions [85] (Fig. 4). In a study of heart failure patients before heart transplantation, the HED uptake in the myocardium closely correlated with uptake 1 and the norepinephrine content, confirming the value of HED imaging as a marker of sympathetic neuronal imaging [86].

A reduction of HED uptake may be a prognostic marker for heart failure patients. Pietilä et al. [87] described HED reduction as an independent risk factor, together with left ventricular end diastolic volume and peak *V*O_2_. An improvement in HED retention was observed after exercise training [88]. Another study compared HED retention with oxidative metabolism, but found no significant association in heart failure patients [89]. Most of these studies using HED seem to give findings similar to those that used MIBG imaging, suggesting autonomic function as an important new parameter for risk analysis and treatment monitoring, independent of other imaging and/or biomarkers.

Since HED PET provides higher spatial resolution and has a higher quantitative capability, it may hold promise for assessing patients with fatal arrhythmias. A global reduction in HED uptake in the myocardium was not observed in patients with Brugada syndrome [90] or those with long QT syndrome [91]. Since most of these studies are preliminary, more clinical studies with high-resolution PET are warranted.

### Adrenoreceptor imaging using PET

**Fig. 5:**
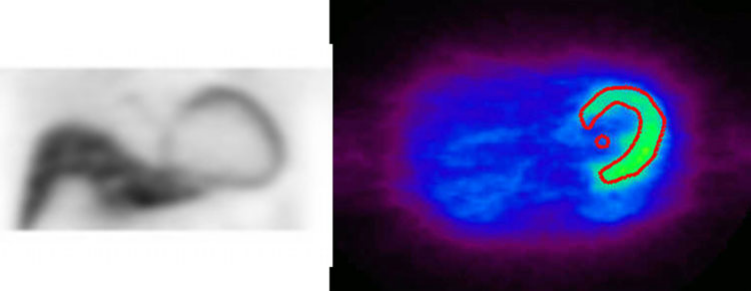
Coronal slice (*left*) and transaxial slice (*right*) of PET images after the administration of ^11^C-CGP 12177 in a patient with idiopathic dilated cardiomyopathy (Color figure online)

Postsynaptic receptors transmit sympathetic signals to target cells. In particular, β1 adrenoreceptor plays an important role in the regulation of myocardial cell function. Compared to the many presynaptic radioligands used for imaging, there are only a few radioligands that are suitable for postsynaptic neuron imaging and probing of neuronal functions. ^11^C-CGP 12177 is a non-selective hydrophilic β-receptor antagonist that provides high-quality PET images with low background activity (Fig. 5). We introduced a modified synthesis method which yielded higher specific activity of CGP 12177 [92]. CGP 12177 binds only to the functional receptors located at the cell surface. Two sequential PET images after an injection of high-specific-activity tracer followed by low-specific-activity tracer allow quantification of β-receptor density using a graphical analysis method [93]. Two other radiolabeled ligands were introduced for adrenoreceptor study: ^11^C-CGP 12388 for β-receptor imaging [94], and ^11^C GB-67 for α1-receptor imaging [95].

**Fig. 6:**
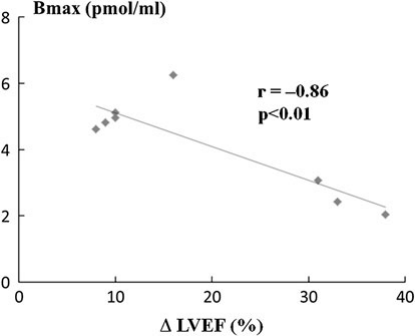
Correlation between pretreatment β-receptor density estimated by ^11^C-CGP 12177 PET and the improvement in left ventricular ejection fraction (ΔLVEF) after long-term β-blocker treatment in patients with idiopathic dilated cardiomyopathy

CGP 12177 has been used to demonstrate β-receptor downregulation in many heart failure patients [96–98]. Our data suggested that myocardial β-receptor density is inversely correlated with the MIBG washout rate and positively correlated with a delayed H/M ratio, indicating that decreased β-receptor density was due to the increased presynaptic sympathetic tone [98]. These results suggest that myocardial β-receptor density may be directly associated with the beneficial response obtained by β-blocker treatment in heart failure patients. In addition, there was a close relationship between myocardial β-receptor density assessed by ^11^C-CGP 12177 PET and the improvement in left ventricular function after long-term β-blocker treatment in patients with idiopathic dilated cardiomyopathy (Fig. 6) [99]. We thus suggested that patients with advanced downregulation of myocardial β-receptor density have higher resting adrenergic drive and may derive greater benefits from the anti-adrenergic effects of β-blocker treatment. Further studies with many more patients with heart failure are warranted to test this finding.

## Conclusions

Cardiovascular molecular imaging allows tissue characterization in myocardial disorders at molecular, subcellular, and cellular levels. It has potential for assessing the severity of heart failure and for monitoring treatment effects. ^123^I-labeled MIBG, as a marker of adrenergic neuron function, could play an important role in the risk stratification of heart failure patients. Presynaptic and postsynaptic neuron imaging using PET can potentially allow better quantifications of autonomic function. More clinical experience is needed to confirm the clinical impact of these imaging modalities for the management of heart failure patients.
